# Damage shielding mechanisms in hierarchical composites in nature with potential for design of tougher structural materials

**DOI:** 10.1098/rsos.181733

**Published:** 2019-03-20

**Authors:** Otte Marthin, E. Kristofer Gamstedt

**Affiliations:** Division of Applied Mechanics, Department of Engineering Sciences, Uppsala University, Uppsala, Sweden

**Keywords:** hierarchical structure, biomimetic, microstructure, shielding, composite, micromechanics

## Abstract

Load-carrying materials in nature, such as wood and bone, consist of relatively simple building blocks assembled into a hierarchical structure, ranging from the molecular scale up to the macroscopic level. This results in composites with a combination of high strength and high toughness, showing very large fracture surfaces indicating energy dissipation by cracking on multiple length scales. Man-made composites instead consist typically of fibres embedded in a uniform matrix, and frequently show brittle failure through the growth of critical clusters of broken fibres. In this paper, a hierarchical structure inspired by wood is presented. It is designed to incapacitate cluster growth, with the aim of retaining high strength. This is done by introducing new structural levels of successively weaker interfaces with the purpose of reducing the stress concentrations if large clusters appear. To test this hypothesis, a probability density field of further damage growth has been calculated for different microstructures and initial crack sizes. The results indicate that the hierarchical structure should maintain its strength by localization of damage, yet rendering large clusters less harmful by weakening the resulting stress concentration to its surroundings, which would lead to an increase in strain to failure. In this context, the potential of using the biomimetic hierarchical structure in design of composite materials is discussed.

## 1. Introduction

**Figure 1.**
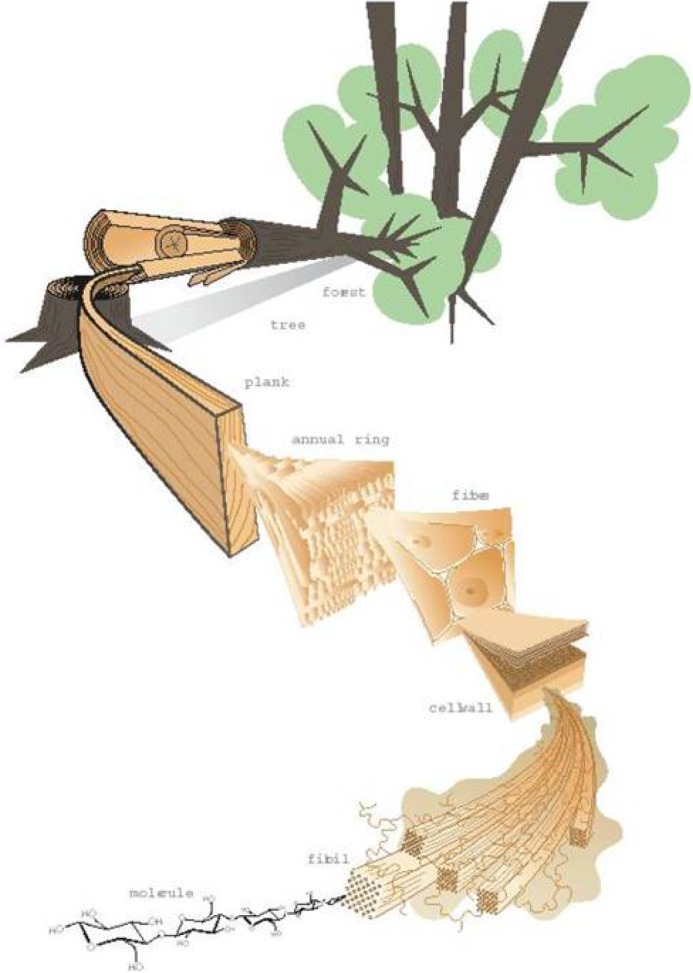
Descriptive image of the hierarchical structure of wood, from [2] adopted by Harrington [3].

Natural load-carrying materials like wood and bone are tough and damage-tolerant [1]. There are some common traits in their material structural, namely a preferred orientation of well-bonded nanoscale fibrils and a hierarchical design for higher length scales with the same preferred orientation along the main loading direction. This can be illustrated for wood with a schematic in figure 1 adopted from Harrington [3]. The same general features also apply for cortical tissue in long bones [4]. The hierarchical structure is most likely not coincidental, but should have been developed by evolution for structural purposes. Furthermore, on each hierarchical level, from elementary microfibrils, over microfibrils, tracheids, annual rings up to the macroscopic grain level, the oriented features are more weakly bonded to each other. At the nanoscale, the elementary microfibrils are tightly bonded to each other by hemicellulose, with a shear strength roughly estimated to 100–200 MPa [5], creating a microfibril. These are in turn placed in a matrix of mainly lignin, whose shear strength has been estimated to about 20 MPa [6], and constitutes the cell wall of tracheids. These values should then be compared to the relatively weak strength of the bonding between wood cells, here approximated by the macroscopic shear strength of solid wood of around 5–12 MPa [7]. Although the strength values at various scales can only be considered as rough estimates, the increasing shear strength at decreasing length scales can be established for biological structural materials like wood. Such an organization results in different types of stress redistribution around breaks of different sizes, hence affecting the type of failure being ductile or brittle [8]. Simultaneous damage and crack growth at different length scales often results in large fracture surfaces, thereby increasing the material toughness [9], yet still achieving a relatively high tensile strength.

The natural fibre composites are made of relatively simple building blocks, like cellulose, lignin and hemicellulose for the case of wood, and collagen and minerals for bone [10]. These constituents do not possess any significant mechanical performance in their neat form on a macroscopic bulk scale. Yet, as a natural material with their inherent composition and structure on several scales, they show a remarkable tensile strength and toughness in the direction they are meant to carry load. This phenomenon is exemplified for bone by Fratzl & Weinkamer [1].

In contrast with natural materials, man-made composites are normally made with one type of fibre embedded in a uniform polymer matrix. For the extreme case of uniaxial tensile loading, high-performance fibres, typically carbon fibres, are mainly oriented in the load direction and surrounded in a more ductile matrix, serving to redistribute the load around broken fibres to the adjacent intact fibres. To improve the stress transfer between fibres, with the aim of improved strength, lots of research has been done to increase the interfacial strength between the fibres and the matrix. This leads to more localized stress fields which under certain conditions may result in higher strength through restricting the possible propagation of damage to small regions around existing breaks, e.g. [11]. The downside is that new fibre breakages with high probability will happen close to an existing break, effectively forming clusters of broken fibres. Although smaller clusters may be safely contained, there exists a maximum cluster size where they start to grow unstably, referred to as a critical cluster, resulting in a brittle failure. Owing to its importance to composite strength, considerable research has been carried out on the subject of cluster growth and critical cluster size, both simulations [12,13] and experimental investigations [14,15]. Increasing the number of structural levels by introducing tough interlayers in laminated composites has already been seen to increase overall toughness in other load cases [16,17]. By understanding how the structural design on various levels in natural fibrous materials enhances the mechanical performance, new stronger and tougher fibre composites may be designed.

**Figure 2.**
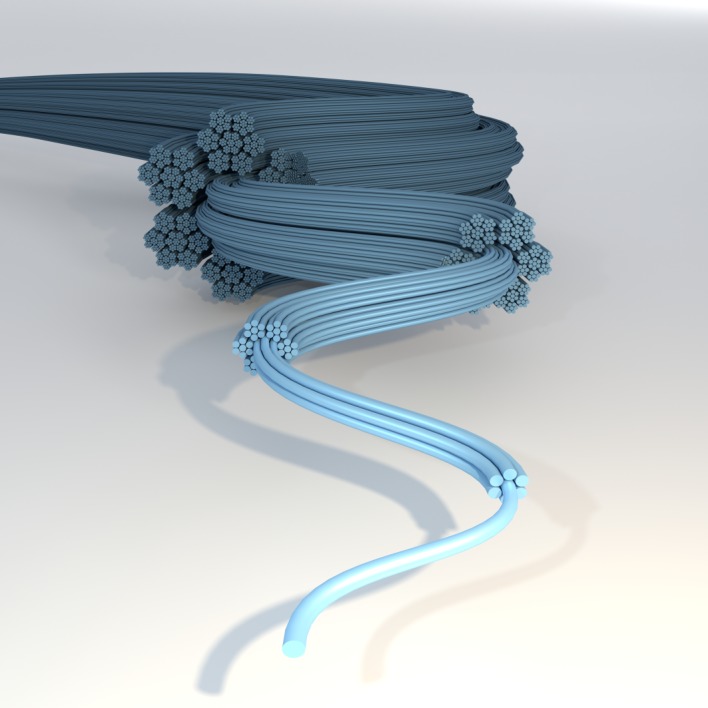
Hierarchical structure of a model composite, where each fibre bundle is bonded slightly more weakly for each larger structural level.

In this paper, the hypothesis that will be tested is that the hierarchical structure of a fibre composite enhances toughness. The toughness considered here is the resistance to further damage growth, i.e. further fibre breakage. This will be done by exploring a shielding effect due to a hierarchical structure using numerical simulation of discrete fibres with successively weaker interfaces. Figure 2 schematically illustrates a hierarchical fibre composite, where the interface between fibre bundles is stronger at smaller length scales and weaker at larger length scales. It will be illustrated how a weaker interface on a larger level can incapacitate the formation of critical clusters that would otherwise lead to ultimate failure. Although a natural hierarchical material usually involves many length scales (e.g. elementary microfibril, microfibril, fibre, earlywood/latewood, sapwood/heartwood, etc.), we confine ourselves here to only two scales to eliminate unnecessary degrees of freedom for the study of the shielding effect. Focus is placed on the formation of new fibre breaks close to the existing fibre breaks, where a hierarchically designed structure is compared with a uniform composite structure. Full simulation of accumulating fibre breaks up to ultimate failure and strength predictions would be the next natural step, but is not the scope of this presentation.

First, some key methods used to simulate failure in fibre composites will be outlined, followed by the reason for the method chosen here and how it has been modified to serve the specific purposes in this work. The fibre strength statistics are then introduced, together with their numerical implementation and what quantitative results one may gather from them. The Methods section is then finalized by defining the geometry of the simulations and necessary parameters for the analysis. In the Results section, the outcome of the simulations is presented and their implications and accuracy are discussed. Finally, some conclusions will be drawn from this work with emphasis on how the observed shielding effects could be used in the design of man-made composites.

## 2. Methods

### 2.1. Numerical approaches

Micromechanical modelling of composite materials to estimate strength and toughness has been a widely-studied research field over the last 60 years or so. One very popular approach is the shear-lag theory introduced by Cox [18]. In shear-lag in fibre composites, the matrix is not considered explicitly, but implemented as the coupling forces between fibres, and fibres are assumed to have an infinite effective shear stiffness. Hedgepeth [19] derived an analytical solution of the stress field around a fibre break based on the shear-lag assumption. Some of the key modelling investigations of relevance to the present situation will be briefly outlined in the following. What they have in common is that they build upon the shear-lag theory and try to approximately solve

2.1 $$A_{i}\frac{d\sigma_{i}}{dz}=-\underset{j}{\sum }h_{i}^{j}\tau_{i}^{j}\;,$$

where *σ_i_* is the stress in fibre *i*, *A_i_* its area, *z* is the coordinate in the fibre direction, *τ_i_^j^* is the shear stress acting on fibre *i* from its *j*th neighbour and *h_i_^j^* is some weight of the interaction, together with the boundary condition that broken fibres do not carry any stress in the crack plane. The analytical solution [19] was later transferred to three dimensions by Hedgepeth & van Dyke [20], where fibres are connected only with their nearest neighbours. Landis *et al*. [21] added connectivity also to the next nearest neighbours using the finite-element method (FEM) and then solved the problem analytically. This latter solution has been shown to give similar stress fields as given by detailed FEM simulations, e.g. [22]. However, the analytical solutions are restricted to the case of a single break. By the use of FEM [21] or finite differences [23], equation (2.1) may be solved numerically and hence provide solutions also for more complex fracture patterns. The simulations, however, quickly become time-consuming when the composite grows. To counteract this, a break-influence superposition scheme was developed by Sastry & Phoenix [24], where the stress field was calculated by interpolations of the analytical solution for a single break. Beyerlein *et al*. [25] later included the treatment of an elastoplastic matrix in the scheme. To further reduce the computational cost, Zhou & Curtin [26] developed a Green's function method, where the stress field in the plane, normal to the fibres, of a single fibre break was known and the stresses were assumed to change linearly from the crack plane along a predefined length. More recently, Pimenta & Pinho [27] developed a model that, by recursive transfer of stress between two fibres, straightforwardly calculates strength distributions of composites consisting of several thousands of fibres. Furthermore, by the use of fibre bundle models, e.g. [28], the computationally achievable volume is greatly increased compared to shear-lag theory, in addition to enabling a rational procedure of simulating composites with random fibre arrangements. However, to do so, the stress field around breaks are assumed to have a geometry-independent form, from which the stress redistribution may be derived.

In this work, a hierarchically structured composite with two types of matrix materials on different scales will be investigated. This situation is presently not entirely compatible with the above-mentioned key approaches. The computationally elastoplastic break-influence superposition approach [25] is based on the analytical elastic solution, and may therefore in principle be used in this. However, it currently supports two-dimensional composites, whereas we would like to address the three-dimensional situation. As Green's function method [26] requires a predefined relaxation length, which depends on the constitutive behaviour of the matrix, it is therefore not directly applicable in this problem due to the multiple matrices on different length scales. The recursive procedure by Pimenta & Pinho [27] is an appealing solution, although due to the assumption that stress is shared between two fibres, it is probably better for studying the effect of size scaling on composites than to estimate the effects from different hierarchical microstructures in a full three-dimensional situation. The fibre bundle models [28] are useful in studying the effects from different geometries when a geometry-independent form of the stress redistribution exists. In a single elastic matrix material, this is arguably the case, however not when the matrix material varies in space.

The presented numerical approaches to model stress development and fibre breakage in unidirectional composites all have their special advantages and limitations. Rather than implementing one of these approaches, it is suggested to take one step back and use a finite-difference scheme to solve equation (2.1), which allows for flexibility regarding unconventional geometries like the present hierarchical two-matrix structure. The downside is the computational cost, which limits the number of fibres in the simulations.

### 2.2. Modelling

To solve the system in equation (2.1) numerically, a modified version of the finite-difference scheme first introduced by Oh [23], and later extended to three dimensions with nonlinear constitutive models by Okabe *et al*. [29,30], was used. In their method, the fibres are placed in a square or hexagonal arrangement and they are only connected to their closest neighbours. In the case of a square arrangement, this results in the weight *h* = *πr/*2, where *r* is the fibre radius. The fibres are then discretized using finite differences, while the shear in the matrix between fibres is described by shear springs. Both plasticity and debonding between fibres and matrix are treated in their model, although the debonding length is given as an input parameter. To the knowledge of the authors, plastic deformation is, however, not considered.

In our work, the finite-difference model has been generalized to include both multiple matrix materials and plastic deformation. The matrix materials are assumed to be linear elastic-perfectly plastic, which have been shown to be a good approximation for some composites by van den Heuvel *et al*. [31], who estimated the stress profiles near a fibre break using micro-Raman spectroscopy. The approximation of the shear in the model is given by

2.2 $$\tau_{i}^{j}=G_{i}^{j}\frac{u_{i}^{j}-u_{i}-u_{p_{i}^{j}}}{d}(1-P_{i}^{j})+\tau_{y}i^{j}P_{i}^{j}\zeta_{i}^{j}\;,$$

where *u_i_* and *u_i_^j^* are the displacement of fibre *i* and its *j*th neighbour, *u*_p_*i*__^*j*^ is the plastic displacement between them, *G_i_^j^* and *τ_yi_^j^* are the shear modulus and shear yield stress in the matrix between the fibres, *P_i_^j^* is 1 if the matrix is plastic and otherwise 0, *ζ_i_^j^* is the sign of the yielded shear and *d* is the distance between the centres of the fibres. Note that the model does not distinguish whether the matrix is yielding or sliding along the fibre with friction. The same treatment, as of *G_i_^j^*, may be used to introduce non-regular fibre arrangements, as well as varying fibre materials, spacing and diameters. This will however not be done here, where the focus is on effects of the two-scale hierarchical structure. Furthermore, due to the treatment of the boundary conditions at broken fibres [23,29], it becomes non-symmetric and hence time-consuming to solve. The scheme was therefore modified to a spring assembly, where the fibres are also treated as a collection of springs. In practice, the only variation on this lies in the treatment of the broken fibres. The effect of this on the stress peaks from a single break was simulated and deemed negligible. This treatment may enable the possibility of non-uniform discretization and variations in fibre diameter within a single fibre, although this also lies outside of the scope of this article. The left-hand side of equation (2.1) is then discretized as

**Figure 3.**
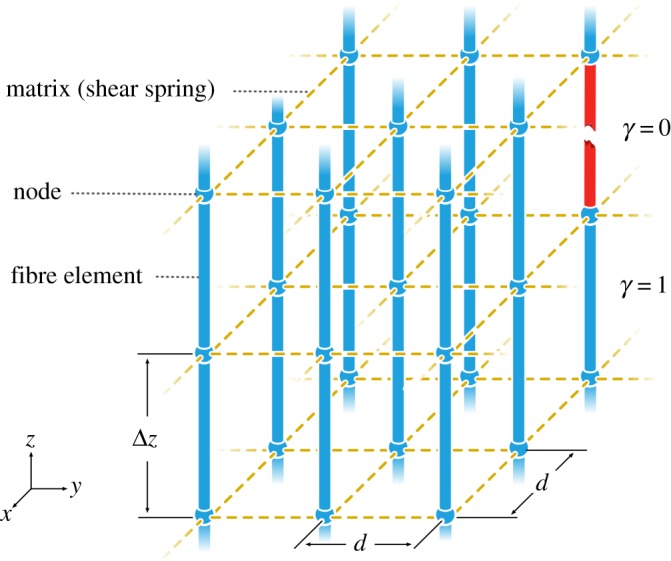
Subset of the discretized square-packed composite, where *d* is the distance to the nearest fibre neighbour, Δ*z* is the length of fibre segment and *γ* indicates if the fibre is intact (*γ* = 1) or broken (*γ* = 0).

2.3 $$\frac{d\sigma_{i}}{dz}(z)\approx E\gamma_{+}\frac{u_{i}(z+\Delta z)-u_{i}(z)}{\Delta z^{2}}-E\gamma_{-}\frac{u_{i}(z)-u_{i}(z-\Delta z)}{\Delta z^{2}},$$

where *E* is the elastic modulus of the fibre, *Δz* is the length of the fibre springs and *γ*_+_ and *γ*_–_ correspond to whether the fibre spring above, or under the node, respectively, are intact, 1, or broken, 0. A subset of the discretized composite is represented in figure 3.

Load is applied to the composite using Dirichlet boundary conditions in both ends of the composite, which are slowly moved away from each other. The system is solved using the trapezoidal method, with each step solved for using the Newton–Raphson method. It was found that breaking fibres may induce oscillations in plasticity of the shear springs. To reduce this numerical artefact, it was necessary to break the fibres slowly by reducing *γ* gradually from 1 to 0, solving the system to convergence for each step.

To implement a hierarchical structure of a composite, the fibres were divided into bundles. The fibres in a bundle were connected using one type of matrix. However, the connection between fibres within different bundles consisted of another type of matrix with a higher shear strength. This way, two structural levels were achieved, one within the bundles and one between bundles. In general, one may introduce bundles of bundles, resulting in yet another structural level, and so on. In this way, a comprehensive hierarchically structured composite could be achieved, as depicted in figure 2, which is inspired by the structure in biological materials such as wood portrayed in figure 1. For a well-defined numerical investigation with a limited number of parameters, the hierarchy is presently limited to only two levels.

### 2.3. Fibre strength statistics

The failure strength of fibres is mainly attributed to randomly located flaws. This results in longer fibres having lower strength due to their larger volumes and hence higher probability of containing a sufficiently large flaw. This type of scaling of strength with size is referred to as weakest link scaling, introduced by Peirce [32]. There are several ways to implement weakest link models in composite simulations. A popular method, usually referred to as a Monte Carlo simulation, is based on the assignment of strength to fibre segments according to a given distribution. This is done prior to solving the system, and as the simulation continues, broken segments are continuously removed. The Monte Carlo simulation of accumulating fibre breaks up to the point of final failure is a formidable exercise and does not fit into this presentation, which is limited to the effects of a hierarchical structure on the underlying stress shielding and probability of subsequent fibre failure. Therefore, the strength distribution is used directly to estimate probabilities of failure.

If the mean stress over a fibre segment, i.e. a fibre spring element, is known, the probability of failure is approximated as the probability that a random number from the strength distribution is lower than the mean stress

2.4 $$F_{\Delta z}(\sigma )=P(X\leq \sigma ),$$

where *F*_Δ*z*_*(σ)* is the probability that a Δ*z* long fibre breaks under the stress *σ* and *X* is the random variable from the strength distribution. Furthermore, the probability that a fibre will fail somewhere is the probability that not all fibre segments will survive under their stress, i.e.

2.5 $$F_{L}(\sigma )=1-\underset{i}{\prod }(1-F_{\Delta z}(\sigma_{i}))\;,$$

where the fibre length is *L* and its *i*th segment is subjected to a stress of *σ_i_*. Similarly, the mean distance in the *z* direction from an existing break to a potentially new one was calculated, weighted by the probability of failure

2.6 $$\langle z\rangle =\frac{\int |z|F_{\Delta z}(\sigma (z))dz}{\int F_{\Delta z}(\sigma (z))dz},$$

where the existing fibre break or breaks are located at *z* = 0. In this work, the interest lies in how a cluster of broken fibres affects the probability of failure in its surrounding intact fibres. The probability of failure in the situation without a fibre break is therefore not of interest. This can be dealt with in equations (2.4)–(2.6) by assuming that all fibre segments survive the nominal stress before the fibre break. The probability that a fibre segment breaks under the stress, *σ* assuming it has survived a nominal stress *σ*_0_, is then given by

2.7 $$F_{\Delta z}^{{\;}^{\prime }}(\sigma )=1-\frac{1-F_{\Delta z}(\sigma )}{1-F_{\Delta z}(\sigma_{0})}.$$

Furthermore, the mean distance from the break to the next will be estimated, calculating the components in the longitudinal and transverse directions separately. This can be achieved by first calculating the probability of each fibre failing, using equation (2.5). The mean distance in each component is then calculated by

2.8 $$\langle z\rangle =\frac{\sum_{j}\langle z_{j}\rangle F_{L,j}}{\sum_{j}F_{L,j}},$$

where 〈*z*〉*_j_* is the mean distance from fibre *j* and *F_L,j_* is the probability that fibre *j* will break. Note that in the case of the longitudinal component, 〈*z*〉*_j_* is first calculated in equation (2.6), while the transverse component of the distance is constant along a fibre.

Finally, in the classical works of Hedgepeth [19] and Landis *et al*. [21], among others, the variables are neatly expressed in dimensionless form. To make our results comparable with theirs, stresses are normalized to the nominal stress and the longitudinal dimensions are normalized according to

2.9 $$\xi =\sqrt{\frac{2r}{d}}\sqrt{\frac{G}{E}}\frac{z}{2r},$$

where *z* is the longitudinal position and *G* is the shear modulus of one of the matrix materials in the case where different elastic properties are used. It can be noted that this transformation was derived principally as suggested by Hedgepeth & van Dyke [20], using the weight *h* = *πr*/2 as given by Okabe *et al*. [29] and written in a form similar to that of Landis *et al*. [21].

### 2.4. Simulations

**Figure 4.**
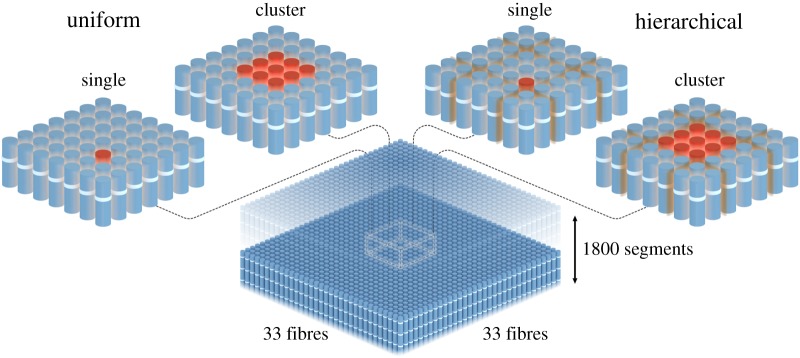
Geometry of the four cases used during simulations, from left to right: uniform structure-single break, uniform structure-cluster break, hierarchical structure-single break and hierarchical structure-cluster break.

To show the effect a hierarchical structure can have on damage propagation, an example composite is used, consisting of 33 by 33 fibres, each divided into 1866 segments. These numbers are a compromise between computational cost and a sufficient large composite to minimize edge effects. The hierarchical composite consists of 11 by 11 bundles of 3 by 3 fibres, as seen in figure 4 for a cross-section. The probabilistic fibre breakage is evaluated for two cases of breaks; the first case is a single broken fibre, and in the second case, a whole bundle has broken and created a cluster, as shown in figure 4.

The chosen parameters defining the composite are compiled in table 1. Specifically, *τ_y_* corresponds to the matrix shear yield stress or a corresponding frictional shear stress of the weak interface. The interfacial shear strength of the strong interface is assumed to be infinite, i.e. no shear plasticity or frictional debond sliding is allowed. The chosen composite length was large enough to avoid effects from the longitudinal boundaries, and then balanced with realistic values for loading and matrix failure, i.e. the nominal strain and shear yield stress. The statistical analysis is based on the two-parameter Weibull distribution, commonly used for both man-made fibres, e.g. [13], and natural fibres, e.g. [33]. The cumulative Weibull distribution is given by

**Figure 5.**
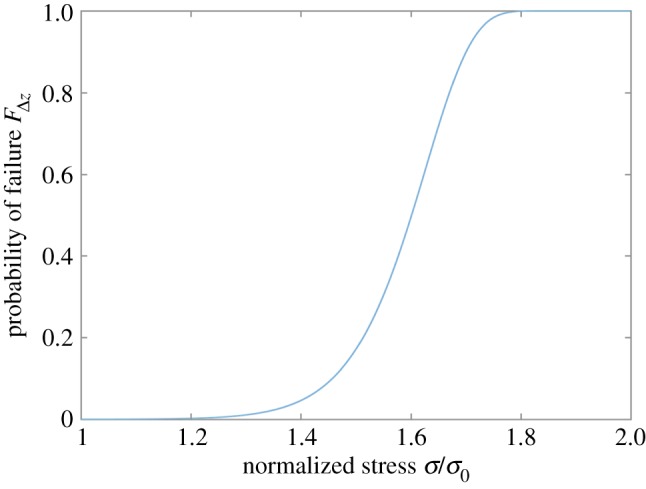
Probability of failure of a fibre segment, given by a Weibull cumulative density function with respect to normalized strength. The fibre segments are all assumed to have survived the nominal stress, *σ*_0_.

2.10 $$F(\sigma )=1-\exp\left(-{\left(\frac{\sigma }{\sigma_{m}}\right)}^{\rho }\right),$$

where *σ*_m_ is the scale parameter and *ρ* the shape parameter. The values of the Weibull parameters (cf. table 1) were somewhat arbitrarily set to resemble typical fibre strength distributions with a relatively large variability. The resulting strength distribution of the fibre segments is shown in figure 5, where the strength has been normalized with respect to the nominal stress. Under the given nominal stress, this results in a mean number of failures of 115 in the test volume of 33 × 33 fibres, 35 mm long.

**Table 1.** Chosen parameter values in the simulations: Dimensions, mechanical properties and Weibull strength distribution.

composite length	length normalization	applied strain	shear yield strain (weak)	normalized scale parameter	shape parameter
*L*	$\sqrt{\frac{2r}{d}}\sqrt{\frac{G}{E}}\frac{1}{2r}$	*ɛ*	*τ_y_/G*	*σ*_m_/*σ*_0_	*P*
3.5 × 10^−3^ m	1.5513 × 10^4^ m^−1^	0.03	0.0124	1.631	20

## 3. Results and discussion

**Figure 6.**
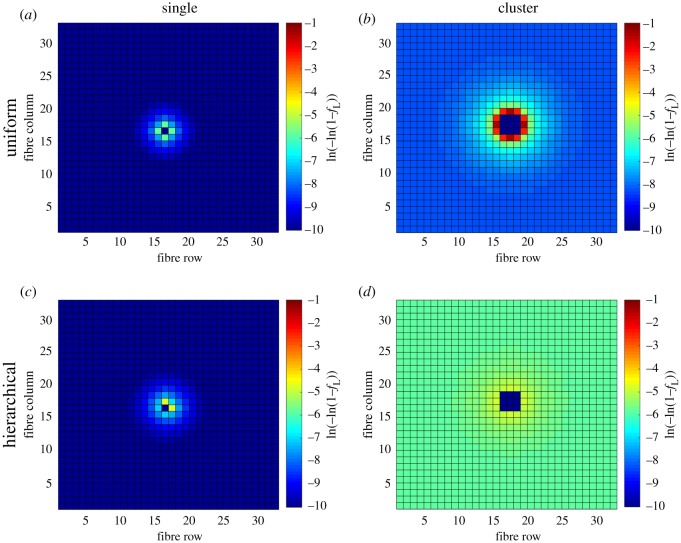
Probability density of failure of fibres in the composite for the four cases: (*a*) uniform structure-single break, (*b*) uniform structure-cluster break, (*c*) hierarchical structure-single break and (*d*) hierarchical structure-cluster break.

The shielding effect from the hierarchical structure can be shown by the probability density of failure of fibres in figure 6, where it is assumed that all fibre segments survived the nominal stress, given by equation (2.7). For convenience in comparing the four different cases, their probabilities of failure are represented as ln(–ln(1–*F*)) as commonly used in Weibull plots [34]. In the case of a single break, the hierarchical structure limits the options for further fibre breakage, figure 6*c*, compared to the uniform structure in figure 6*a*, confining the higher stress concentrations inside its inner bundle structure. In the case of a cluster of breaks, the uniform structure shows high stress concentrations around the edge of the cluster, as seen in figure 6*b*. The hierarchical structure instead distributes the stress over a much larger volume, thus lowering the stress concentrations, as can be seen in figure 6*d*. These stress redistributions mean that fibre failures in the uniform structure have a higher probability of increasing the cluster size, while this is avoided in the hierarchical structure by a delocalization of the stress concentration.

The qualitative representation in figure 6 of shielding in a hierarchical structure is also quantified in some indicative numbers in table 2. Here, the mean transverse distance, *r*, from a new break to the centre of the existing break normalized to the distance between fibres is presented. The result shows that when the break grows from a single fibre break to a cluster contained in a bundle, the mean transverse distance reduces in the case of a uniform structure, while it increases with a hierarchical one. The latter trend can be attributed to a boundary effect, which can be observed in figure 6. The cross-section of the simulated composite is not large enough for the stress field to become uniform and equal to the applied nominal stress far away from the broken fibres. The low probability of failure and stress levels close to the nominal stress (represented by a dark blue colour) for the single fibre breaks in figure 6*a,c* is not found for the cluster breaks in figure 6*b* and in particular in the hierarchical case in figure 6*d*. Although the computational cost was too high in the present case, the results indicate that the composite cross-section should be more than one order of magnitude larger than the largest hierarchical structure to avoid boundary effects that effectively increase the probability of failure of the intact fibres.

**Table 2.** Calculated quantities for comparing the four cases with uniform and hierarchical structure with single or cluster fibre breaks. The distance 〈*r*〉/*d* is the mean transverse distance between a new and pre-existing break(s) normalized to the distance between fibre centres, 〈*ξ*〉 is the dimensionless mean longitudinal distance between new and pre-existing break(s) and 〈*N*_f_〉 is the mean number of new fibre breaks in the whole composite volume caused by the inserted fibre break(s).

	uniform		hierarchical	
	single	cluster	single	cluster
transverse break distance, 〈*r*〉/*d*	6.9	3.9	6.5	12.0
longitudinal break distance, 〈*ξ*〉	1.1	0.4	1.0	3.2
number of fibre breaks, 〈*N*_f_〉	0.0	1.9	0.1	3.3

**Figure 7.**
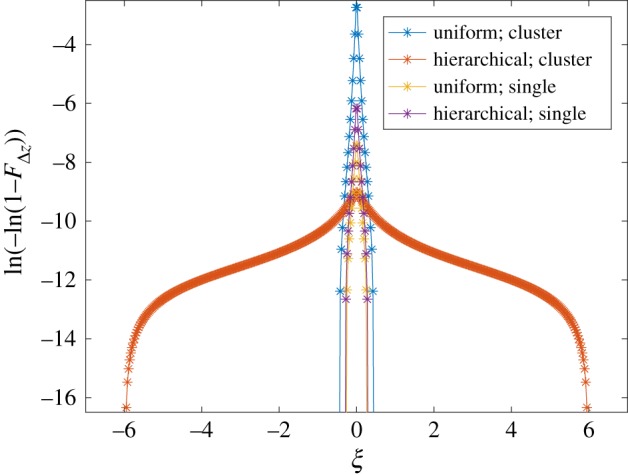
Profiles of the probability of failure along the nearest intact fibre in the four cases. The probability of failure for each segment is given by ln(–ln(1–*F*_Δz_)) and plotted along the dimensionless longitudinal coordinate *ξ*, where *ξ* = 0 represents the crack plane. Note that the full longitudinal dimension of the composite is approximately –27 < *ξ* < 27.

The probability of failure profiles along the nearest intact fibre with highest probability of failure in each of the four cases are seen in figure 7, where the hierarchical structure is seen to dissipate the damage probability over a larger longitudinal distance. In the case of a single broken fibre, both the uniform structure (yellow) and the hierarchical structure (purple) show a similar trend of a high probability of failure close to the crack plane. In the case of a cluster, the uniform structure still has a localized high peak of failure probability in the plane of the fibre breaks (blue), but the hierarchical structure smears the probability of failure out over a much larger distance (red). If a fibre breaks close to another broken fibre, but the breaks in the two adjacent fibres are at a large longitudinal distance from one another, and the increase in stress concentrations in the crack plane is much smaller than if it had broken close to the original break plane. Also, in the case of failure of the entire composite or coalescence of fibre breaks to form a larger cluster, the broken fibres need to be pulled out from the matrix. Qualitatively, the energy required to pull out fibres is larger if the fibres have broken longitudinally far from each other. In table 2, the mean longitudinal distance from the original crack plane to a new break is presented, where the distance 〈*ξ*〉 is given in the dimensionless form given by equation (2.9). It is seen that in the case of a broken cluster, the mean longitudinal distance to a new break is a magnitude larger for the hierarchical structure than for the uniform structure. This longitudinal spread of fibre breaks would promote energy dissipation by pull-out of fibres, in contrast with brittle failure with a planar crack plane.

Table 2 also shows the mean number of new failures, 〈*N*_f_〉, assuming all segments survive the nominal stress. It is seen that the hierarchical structure has a higher mean number of new failures than the uniform structure. This can be attributed to increased stress concentrations over a much larger volume than in the uniform case, stemming from the boundary effect discussed previously. It can be noted that the mean number of new failures due to the prescribed breaks are considerably smaller than those caused by the nominal stress only.

It should be noted that the present simulations were limited to 33 × 33 fibres due to computational cost. Realistically, a composite member, biological or man-made, would contain significantly more fibres. It remains to be seen if a hierarchical composite design could reduce the propensity of further fibre breakage in large-scale simulations. At the present stage, it has however been shown that the hierarchical case leads to a wider spatial distribution of new fibre breaks due to a delocalization of the stress concentration, when compared with the uniform case. In general terms, spreading of damage is known to increase the toughness of materials compared with localization of damage. It can be argued that a microstructure consisting of relatively weakly bonded bundles (left in figure 4) should be suitable to incapacitate clusters of fibre breaks just prior to becoming critical. The optimal number of fibres in each bundle would then depend on the distribution of fibre strength, stress transfer between the fibres and size of the composite.

Although attempted, the authors have not succeeded in conclusively showing an increase in macroscopic composite strength due to a hierarchical structure using Monte Carlo simulations with accumulating fibre breaks. A plausible reason for this is the mentioned transverse boundary effects, where the limited cross-sections in the simulations do not allow the stress field to become uniform away from the stress concentration caused by the fibre breaks in the hierarchical structure.

In this context, the dynamic effects can also influence the outcome of simulations to predict ultimate strength. As a fibre or a set of fibres ruptures, a dynamic stress wave emanates from the rupture site. The transient peak stresses in the nearby fibres are certainly going to be higher than the steady-state stresses under quasi-static conditions leading to increased fibre breakage [19], which has been suggested by experiments [14]. These instantaneous overloads were not considered in the analyses presented in reviews of numerical models above. There is reason to believe that the dynamics stress concentration will be larger in the uniform elastic case than for a hierarchical material, where multiple weaker interfaces can deflect and dissipate stress waves more efficiently. The strength of a hierarchically structured composite could then be enhanced further in the more realistic case of dynamic fibre breakage. For obvious reasons, available modelling work is limited to static conditions, because dynamic modelling would be much more difficult requiring, for example, viscoelastic or viscoplastic behaviour of the constituents. Promising work using high-resolution X-ray micro-computed tomography [14,35] show that it is possible to detect the accumulation of single fibre breaks inside unidirectional composite layers, which can provide data to better estimate the stress concentrations around broken fibres in reality, thereby including dynamic and viscoelastic effects. An evident design parameter to tune the stress concentration is the fibre–matrix interface. Especially for brittle composites, such as ceramic–matrix composites, the potential for interfacial design to improve toughness is clear [36].

The benefits of a hierarchical structure could in principle be implemented for man-made unidirectional composites. Incidentally, the hierarchical features found in, for example, wood can be found in rope, a man-made fibrous material where the stress transfer on different scales of the fibrous yarns and strands are controlled by the degree of twist. The number of fibres in each bundle and the twist have been tuned by experience of generations of ropemakers to combine high strength and toughness, mostly by empirical trial and error and experience, and only more recently based on quantitative mechanical design [37]. In fact, the twist presents itself in the microfibrillar angle in wood materials, and the layered lamellar cell-wall structure can provide some additional toughness when compared with the unidirectional composite configuration [38]. For composites with unidirectional untwisted fibres, a manufacturing method based on pultrusion could be viable [39], in which fibres are pulled through an impregnating resin bath followed by consolidation in a curing stage. If this process is repeated with successively larger assemblies of comprising fibre bundles and successively weaker matrix resins, the envisaged hierarchical composite could be manufactured. How to optimize this process with regard to strength and toughness requires further research and empirical testing.

## 4. Conclusion

In this work, a bioinspired microstructure has been presented with the ultimate goal of combining high strength with toughness by making the composite less sensitive to critical clusters. A hierarchical structure with successively weaker interfacial strength (two scales) has been conceived with the aim of decreasing the stress concentrations in neighbouring large clusters. It has been shown that for single fibre breaks, the presented microstructure statistically limits the propagation of damage to a smaller volume than achieved by simply a uniform structure of high interfacial strength. Yet, when a large cluster of breaks exists, the hierarchical structure was shown to spread out the stress to a larger volume, lowering the probability of further cluster growth. This suggests that the hierarchical microstructure localizes and confines small damages, a process minimizing the probability of the damage spreading. The hierarchical microstructure also makes large clusters less harmful by attenuating their high stress concentrations, suggesting a more ductile and damage-tolerant behaviour. Furthermore, it was shown that the hierarchical structure additionally spreads out the probability of failure in longitudinal direction, at least after the failure of a bundle. A material failing in such a manner usually requires extensive work to pull out the fibres from the surround matrix, suggesting that the hierarchical structure increases the general toughness of the composite. Although the presented results have not yet been successfully reproduced in terms of ultimate strength in Monte Carlo simulations, the observed mechanisms suggest that the hierarchical microstructure may combine high toughness with high strength. The hierarchical structure could in principle be implemented in manufacturing of synthetic composites, inspired by production processes of ropes, albeit without twisting.

## Data accessibility

For the sake of completeness, a MATLAB code is available at: http://dx.doi.org/10.5061/dryad.60vk297 [40]. The code is a direct implementation of the model as described in the paper, using the same symbols and supplied with comments with definitions, etc. The subroutines and functions, called from the main code, are stored in a separate folder.
